# Tracking visual search demands and memory load through pupil dilation

**DOI:** 10.1167/jov.20.6.21

**Published:** 2020-06-26

**Authors:** Moritz Stolte, Benedikt Gollan, Ulrich Ansorge

**Affiliations:** Faculty of Psychology, University of Vienna, Austria; Research Studios Austria, Vienna, Austria; Vienna Cognitive Science Hub, University of Vienna, Austria

**Keywords:** pupil response model, attentional pulses, visual working memory, visual search, online modeling

## Abstract

Continuously tracking cognitive demands via pupil dilation is a desirable goal for the monitoring and investigation of cognitive performance in applied settings where the exact time point of mental engagement in a task is often unknown. Yet, hitherto no experimentally validated algorithm exists for continuously estimating cognitive demands based on pupil size. Here, we evaluated the performance of a continuously operating algorithm that is agnostic of the onset of the stimuli and derives them by way of retrospectively modeling attentional pulses (i.e., onsets of processing). We compared the performance of this algorithm to a standard analysis of stimulus-locked pupil data. The pupil data were obtained while participants performed visual search (VS) and visual working memory (VWM) tasks with varying cognitive demands. In Experiment 1, VS was performed during the retention interval of the VWM task to assess interactive effects between search and memory load on pupil dilation. In Experiment 2, the tasks were performed separately. The results of the stimulus-locked pupil data demonstrated reliable increases in pupil dilation due to high VWM load. VS difficulty only affected pupil dilation when simultaneous memory demands were low. In the single task condition, increased VS difficulty resulted in increased pupil dilation. Importantly, online modeling of pupil responses was successful on three points. First, there was good correspondence between the modeled and stimulus locked pupil dilations. Second, stimulus onsets could be approximated from the derived attentional pulses to a reasonable extent. Third, cognitive demands could be classified above chance level from the modeled pupil traces in both tasks.

## Introduction

In addition to the well-studied pupillary light reflex (the rapid constriction in response to bright stimuli; Loewenfeld & Lowenstein, 1993), the human pupil has been shown to react to a wide variety of cognitive processes, such as attention (e.g., Kahneman, 1973; Karatekin, 2004), emotions (e.g., Bradley, Miccoli, Escrig, & Lang, 2008; Nagai, Wada, & Sunaga, 2002), decisions (Simpson & Hale, 1969), or cognitive load (Hess & Polt, 1964). Moreover, several studies have shown that pupil size increases in response to increased working memory demands (Granholm, Asarnow, Sarkin, & Dykes, 1996; Kahneman & Beatty, 1966), and extensive early work demonstrated that pupil dilation reliably indicates the relative increase in processing demands within a task and between tasks (for review, see Beatty, 1982). Crucially, in contrast to performance measures such as accuracy or reaction time (RT) measurements (which require manual responses), tracking pupillary dilation and constriction imposes no additional task requirements on the cognitive process under investigation but yet at the same time allows monitoring of the currently deployed processing resources (Hyönä, Tommola, & Alaja, 1995).

In theory, using a wearable eyetracker to measure pupillary responses would therefore allow the continuous online tracking of cognitive demands imposed by different tasks and situations (e.g., during reading and writing, human–machine interactions, tool use, or even mere thinking). During these everyday activities, little is known in advance about the exact point in time at which humans begin their cognitive processing, and often there are no available overt performance measures reflective of such processing. Pupil size measures could possibly allow the development of a common metric to compare demands across such situations. However, hitherto, no experimentally validated online algorithm exists that would allow us to successfully classify the demands imposed by different tasks on the basis of measured pupil sizes alone, without prior knowledge of task onsets. Yet, a validation of such an algorithm by laboratory experiments is desirable, as only an experimental approach allows the necessary high control over the independent variables for relatively clear conclusions regarding whether or not a measured pupil response reflects cognitive demands. Therefore, in the present study, we validated a continuously operating online algorithm that does not require prior knowledge of the time at which a demanding process begins in two controlled laboratory experiments. The experiments allowed exact control over stimulus presentation and, hence, control over when our participants could have begun their cognitive processing. As a consequence, in such an experiment, we can also check in a second step, if a continuously operating algorithm that works without knowledge of the time points at which stimuli are presented and that models pupillary responses as events elicited by (retrospectively calculated) attentional pulses (Gollan & Ferscha, 2016) is able to accurately reconstruct the stimulus onsets known to us.

Importantly, when planning our experimental validation, we were careful to ensure ecological validity by mimicking the complexity of cognitive processing in real-world situations at least to some extent. To that end, in Experiment 1, we combined a visual working memory (VWM) task and a visual search (VS) task. In each trial, participants first encoded visual objects into their VWM for a subsequent recognition test. In the retention interval between encoding and probe or recognition display, participants conducted a VS task by searching for a predefined (shape or orientation) target among distractors. This combination of tasks is ecologically valid, as many situations require keeping some information in mind for later usage (e.g., that the telephone number of the project leader has to be included in the final list of telephone numbers of all project employees) while having to conduct visual searches on more or less related information in between (e.g., searching for the telephone number of the project leader on the internet).

We set out to examine the contributions from memory load and perceptual demands to pupil responses and to test whether overall load or demands in a given task can be reliably identified by pupil responses alone. In two experiments, we show interactive, as well as independent, effects of VWM load and VS demands on pupil dilation. In addition to the analysis of task-evoked pupil responses by means of traditional analysis of averages based on stimulus-locked eyetracking data (e.g., Hoeks & Levelt, 1993; Wierda, van Rijn, Taatgen, & Martens, 2012), we also employ mathematical pupil modeling to online monitor cognitive load based on pupil measures alone and without prior knowledge of stimulus or task onsets.

Regarding speed and accuracy of performance, in VS demands on attention are generally estimated from the search slope, a measure of the time to find the target required per each additional distractor item in the display (e.g., Treisman & Gelade, 1980). Efficient search (or pop-out search) usually has a search slope of close to zero (less than 10 ms per item) and is thus barely affected by the number of items in the display, whereas inefficient search (or serial search) usually involves more difficult discriminations and results in steeper search slopes (e.g., Wolfe, 1998). Search efficiency decreases as target and distractors become more similar (e.g., Duncan & Humphreys, 1989). Thus, when searching for the same target under otherwise equal conditions, the search is more difficult (as is evident in a steeper search slope) when distractors more closely resemble the target. Accordingly, if pupil responses indeed track attentional demands in VS then performing the more difficult search should result in stronger pupil dilation compared to the easier search (Irons, Jeon, & Leber, 2017; Oliva, 2019; Porter, Troscianko, & Gilchrist, 2007; but see Porter, Tales, Troscianko, Wilcock, Haworth, & Leonards, 2010). By manipulating search difficulty while keeping physical properties of the stimuli (e.g., luminance, locations, number of items) constant, the results should validate the pupil measurement technique as an indicator for demands imposed by VS. Such results should also confirm that increased dilation corresponds to attentional processing demands even when stimuli are presented very briefly (200 ms) and the task does not require overt eye movements (ruling out load-independent contributions to the pupil dilation measure such as differences due to recording angle of the eye or due to luminance differences at different monitor locations).

Similarly, pupil responses have been shown to indicate increased (visual working) memory demands (e.g., Goldinger & Papesh, 2012; Kafkas & Montaldi, 2011; Kahneman & Beatty, 1966; Kursawe & Zimmer, 2015). Here, we tested whether pupil responses reflect changes in pure VWM load. Moreover, the visual nature of the memory task employed may influence perceptual or memory components of the VS task performed during the memory maintenance interval (cf. Attar, Schneps, & Pomplun, 2016; Horowitz & Wolfe, 1998; Konstantinou & Lavie, 2013; Kristjánsson, 2000; Olivers, Meijer, & Theeuwes, 2006; Olivers, Peters, Houtkamp, & Roelfsema, 2011). Thus, increasing the number of items held in VWM might adversely affect search performance and may be reflected in additional dilatory pupil responses.

## Experiments

In two experiments, we investigated the effects of VS demands and VWM load on behavioral performance and pupil responses. In Experiment 1, VS and VWM load manipulations were combined to test for possible interactions, as both types of demands often occur together in real life settings, but the tasks were performed separately in Experiment 2 to isolate specific contributions. VS demand was manipulated by changing target distractor similarity, whereas VWM load was determined by the number of items to be held in memory. Based on previous literature demonstrating that pupil responses track overall cognitive processing demands, we expected an increased dilatory response to increases in VS difficulty, as well as VWM load, and possibly an interaction when both tasks are performed simultaneously due to shared neural resources.

As we ran both experiments to validate an algorithm for the online detection of VS demand-related or VWM load-related pupil dilations, we also systematically varied background luminance. In this way, we tested whether or not demand or load effects on pupillary responses can be measured under varying ambient luminance conditions, an important question for application of the algorithm outside of the laboratory. Note that the online algorithm allows modeling and subtraction of a concomitantly measured luminance-elicited pupil dilation response. Performance of the online algorithm was compared to more standard calculations of stimulus-locked pupil data.

## Methods

### Participants

Twenty-one observers (nine female) between 19 and 36 years of age (*Mdn* = 22, *SE* = 1.05) participated in Experiment 1, and 17 observers (nine female) between 19 and 27 years of age (*Mdn* = 21.5, *SE* = 0.55) participated in Experiment 2. All participants were recruited from the University of Vienna subject pool, reported normal or corrected-to-normal vision, signed an informed consent before participating, and received course credits for completing the experiment.

### Apparatus and software

The right eye was recorded with a head-mounted, mobile, video-based eye tracker developed by Pupil Labs (Berlin, Germany) (Kassner, Patera, & Bulling, 2014), with a 60-Hz sampling rate (equipped with a world and eye camera; gaze accuracy of 0.6° as estimated by the manufacturer). The eye tracker was connected to a PC running Windows 10 (Microsoft, Redmond, WA) and Pupil Labs software (pupil-labs.com/pupil/) to record pupil size, eye movements, and the external visual surroundings. Stimuli were presented on a 31-cm by 28.5-cm monitor (resolution, 1920 pixels × 1080 pixels; 75 Hz). Experimental stimulus parameters were controlled with Psychophysics Toolbox (Brainard, 1997; Kleiner, Brainard, & Pelli, 2007; Pelli, 1997) and MATLAB 9.3 (MathWorks, Natick, MA). A chin rest was used to support the participant's head relative to the monitor at a viewing distance of 50 cm.

### Design and procedure

In Experiment 1, participants performed a VS task during the maintenance period of a visual VWM task. Memory load (low/high) in the VWM task and search difficulty (low/high) in the VS task varied independently, as well as background luminance (dark/bright). The luminance manipulation was included as a control condition to see if the dilatory response of the pupil can at least be elicited by changing light conditions, as it could be that both of our load manipulations fail to trigger a dilatory response. In addition, the luminance manipulation also allows us to investigate if the dilatory response to load is the same under different levels of ambient luminance, despite the fact that the increase in pupil surface for such a dilatory response is not the same when the pupil is open wide in dark conditions compared to a narrower opening in light conditions. We employed a fully balanced factorial design with three independent variables of two levels each. The task was divided into 24 blocks of 12 trials each. Thus, each unique combination of steps of the independent variables was repeated in three blocks (36 trials total). The order of the blocks was pseudorandomized and counterbalanced across participants.

**Figure 1.**
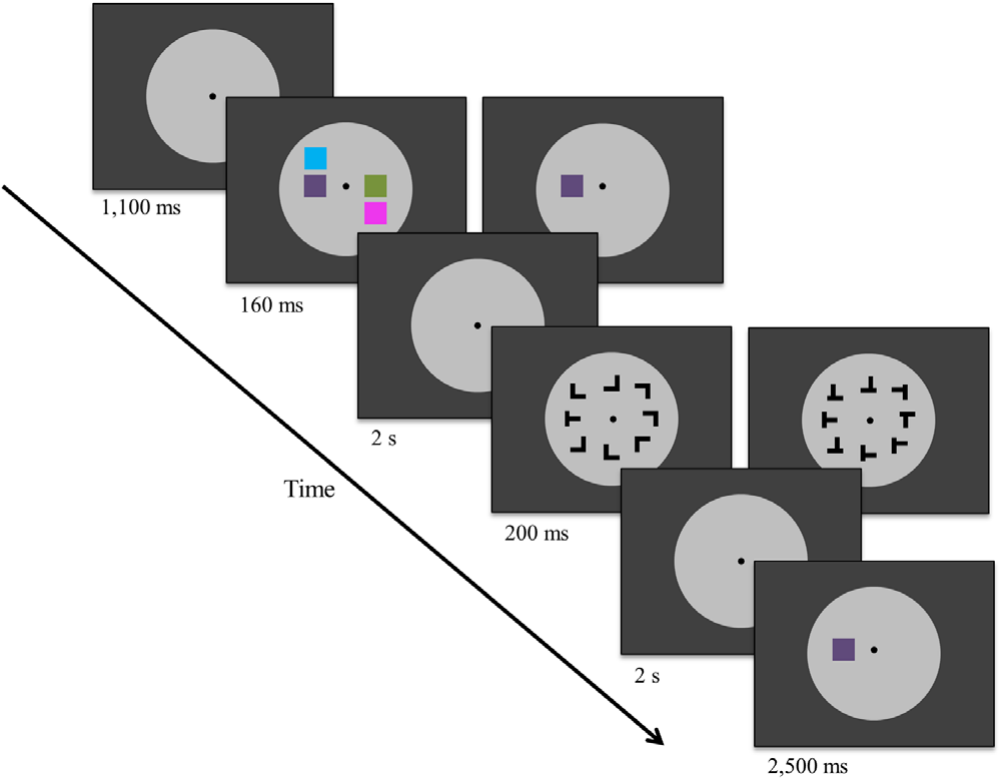
Schematic illustration of trial sequence in Experiment 1 (stimuli not to scale). Examples of trials with high VWM load plus low VS demands (left), and low VWM load plus high VS demands (right). Stimuli were always presented on a gray disk while background luminance varied (same gray as disk or black).

A five-point eye tracker calibration was performed before each experiment. Additionally, during the presentation of the central fixation point a one-point drift check was completed before each trial. All participants were instructed to maintain fixation on a central fixation point throughout each trial. Figure 1 depicts the stimuli and order of events in a trial of Experiment 1. Throughout each block all stimuli were presented on a light-gray (CIE L*a*b* coordinates, 75/–1.3/–2.4) central disk (11.3° of visual angle radius) surrounded by either a black background (0.9/–0.12/–1.9) or a gray background (75/–1.3/–2.4) that filled the rest of the screen. There were two tasks in each trial—a memory task and a search task—with the memory array preceding a search array and with a memory probe display at the end of the trial. In each trial, following a fixation point at the center of the screen (1100 ms), the memory array was presented for 160 ms. The memory array contained either one colored square (low load) or four differently colored squares (high load) subtending 0.57° of visual angle, randomly chosen on each trial from a total of seven different colors of approximately equal luminance—blue (65.10/–6.73/–37.42), green (65.21/–21.35/31.43), turquoise (66.13/–37.34/7.02), pink (64.86/37.41/–6.53), purple (64.78/21.79/–31.10), orange (63.4/31.59/21.15), and yellow (62.36/7.21/37.29)—and randomly placed among nine possible locations within a three-by-three grid (1.8° × 1.8°).

After a blank interval containing only the fixation point (2 seconds), a VS array was presented for 200 ms. The search array consisted of seven distractors and a single target arranged on a virtual circle (radius of 5.15°) equidistant from each other and from fixation. The target was always a T shape rotated 90° left or right (each, 50% of the trials), and it randomly appeared in one of the eight positions. Distractors were L shaped with equal arm length and were randomly rotated 0°, 90°, 180°, or 270°. In the low-load condition of the search task, all distractors were normal L shapes with arms joined together flush at one end; in the high-load condition, one of the arms was slightly offset, making the distractors more similar to the target (Figure 1). Observers made speeded responses within 2 seconds from stimulus onset, indicating the orientation of the target by pressing the left or right arrow key on the keyboard (with the index finger or middle finger of the right hand, respectively). After the response window for the search task, a memory probe (a single colored square) appeared for up to 2500 ms at one of the positions occupied in the original memory array. Observers made speeded responses, indicating whether the color of the memory probe matched the color of the stimulus in the memory array at the same location. The memory probe matched both the location and color of a square in the memory array 50% of the time (it always matched the color of one of the squares in the memory array during the condition of high memory load).

The design of Experiment 1, however, does not allow assessment of the strictly independent effects of each task, as influences from the respective other task cannot be ruled out. In fact, we observed some evidence for a paradoxical facilitation of VS task performance by a higher concurrent VWM load, pointing to perhaps better task performance scheduling under the most difficult conditions—that is, where smart scheduling would be required the most. (An alternative explanation could be that participants sometimes did not perform the high-load VWM task at all, such that the VS performance in these non-VWM performance trials was free from dual-task interference.)

To avoid such complexities in Experiment 2, search and memory tasks were performed separately. Moreover, Experiment 2 addressed a number of limitations in the design of Experiment 1. In the VWM task of Experiment 1, memory load was manipulated by presenting either one or four items. Although there was no indication that luminance itself influenced the pupillary response or task performance under the conditions of Experiment 1, one could argue that the difference in physical stimulation alone may explain the observed differences in pupil size due to differential engagement of the pupillary light reflex (e.g., Kohn & Clynes, 1969). A similar criticism applies to the observed differences in pupil size in response to the memory probe of Experiment 1. Although the memory probe was always a single stimulus, it remained on screen until a key press was made, which occurred on average later in the high compared to the low VWM load condition.

To avoid possible confounds from differences in physical stimulation on pupil responses, stimulus displays in Experiment 2 contained an equal number of stimuli and were of equal luminance for the different levels of demands or load in both tasks. In addition, stimuli remained on screen for equal durations, ruling out differential responses of the pupillary light reflex independent of the level of demand or load. Moreover, the stimulus displays used for the VS task and VWM task were identical, allowing direct comparison between the two tasks. Unlike Experiment 1, search difficulty was manipulated by employing a search asymmetry (i.e., where the search for Target A among Distractors B produced different behavioral results than the search for Target B among Distractors A) (e.g., Treisman & Gormican, 1988) using vertical and tilted lines as targets and distractors. In each trial, participants searched either for a tilted line among vertical distractors (easy task) or for a vertical line among tilted distractors (difficult task) and judged whether a target was present or absent. This task affords a different way to increase search difficulty (presumably relying on different, preattentive processes in early visual cortex than those engaged by target–distractor similarity used in Experiment 1) (e.g., Li, 1999), thus allowing us to generalize the findings from Experiment 1 to other VS tasks and perceptual load manipulations. To effectively demonstrate increased search difficulty in the search asymmetry framework, we used two set sizes in the search display: two and seven items. An increase in RTs for searches among seven items compared to two items (i.e., steeper search slope) for vertical targets (among tilted distractors), but no difference in RTs for the two set sizes (i.e., shallow search slope) when the role of target and distractor were reversed, specifically demonstrates increased search difficulty or higher perceptual demands through less efficient (serial) versus more efficient (pop-out) searches.

Participants performed separate VS and VWM tasks in Experiment 2. The order of the two tasks was counterbalanced across participants. Memory load (low/high) in the VWM task and search difficulty (low/high) in the VS task varied in separate blocks, as well as background luminance (dark/bright). In the VS task, difficulty was manipulated by employing stimuli eliciting a search asymmetry. Specifically, participants were instructed prior to each block to search for either a single vertical line among tilted lines (slower, less efficient search) or a single tilted line among vertical lines (faster, more efficient pop-out search). Set size was either two or seven stimuli, and a target was present on 50% of all trials within a block (and absent in the remaining trials). In the VWM task, the same stimulus arrays (except that here set size was always seven) were used as in the VS task. Memory load in the VWM task was manipulated by instructing participants prior to each block to remember only a single colored line (e.g., “remember BLUE”) or to remember all lines (e.g., “remember ALL”) and then match the color and location of a single memory probe to the original memory array (as in Experiment 1). The VS task was divided into 24 blocks and the VWM task into 12 blocks of 12 trials each. The order of the blocks within each task was pseudorandomized and counterbalanced across participants.

**Figure 2.**
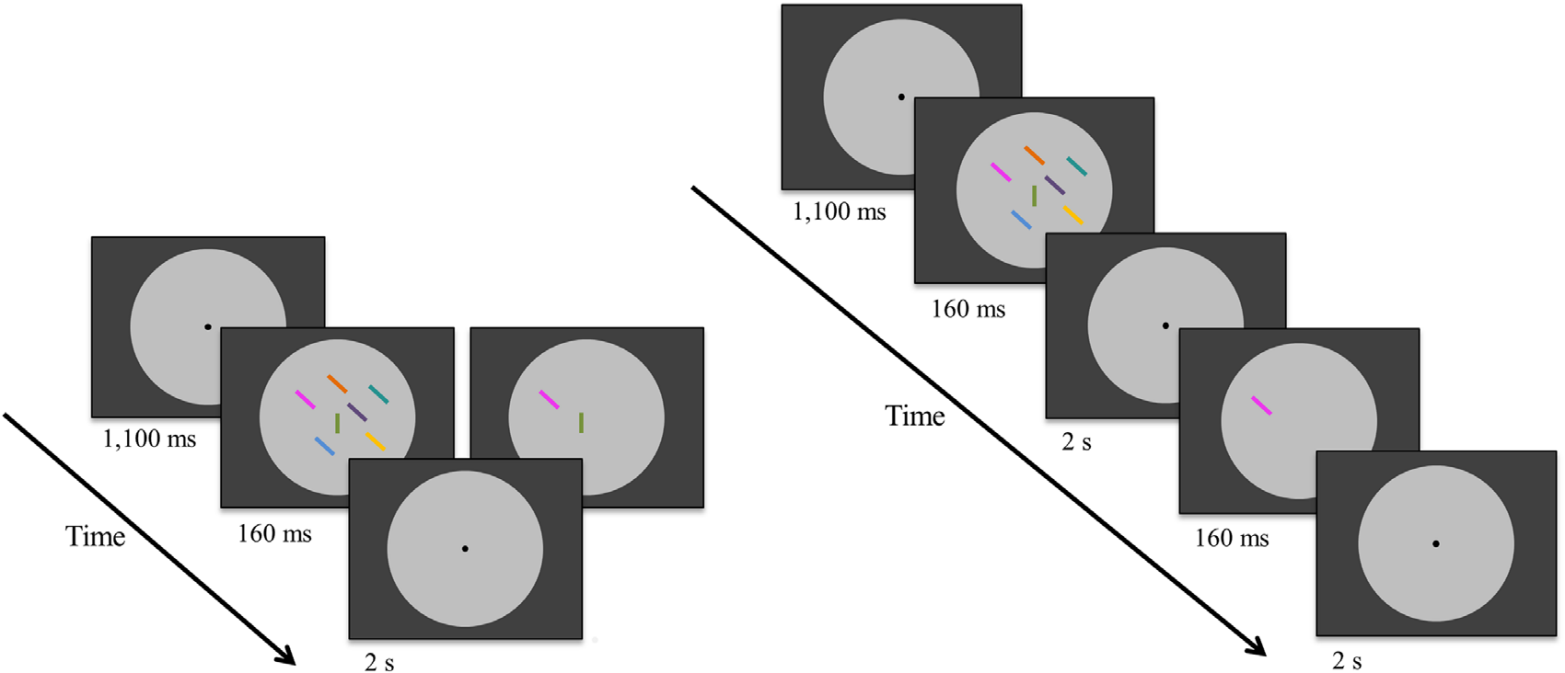
Schematic illustration of trial sequences in Experiment 2 (stimuli not drawn to scale). VS task (left panel) with two or seven items displayed shows an example trial of the high search difficulty condition (less efficient search; the target is a vertical bar that was present on 50% of the trials). In the VWM task (right panel), the memory array looked like the seven-item array in the VS task. Participants matched the location of either a blue probe (one item, low memory load) or a probe of any color (seven items, high memory load). As in Experiment 1, stimuli were always presented on a gray disk while background luminance varied (same gray as disk or black).

Figure 2 depicts the stimuli and order of events for each of the two tasks in a trial in Experiment 2. Throughout each block, all stimuli were presented on a light-gray central disk (radius 11.3 degrees of visual angle) surrounded by either a black or a gray background, as in Experiment 1. This time, the two tasks were realized in separate blocks. In each trial of both tasks, following a fixation point at the center of the screen (1100 ms), an array of equiluminant, colored lines (subtending 0.57°; same colors as in Experiment 1) was presented for 160 ms. In the VS task, the display contained either two or seven lines (in the VWM task, there were always seven lines), randomly placed among 25 possible locations within a five-by-five grid (2.8° × 2.8°) and slightly spatially jittered. The target in the VS task was either a vertical line (among 45° tilted distractor lines) or a 45° tilted line (among vertical distractor lines). Participants made speeded responses within 2 seconds from stimulus onset, indicating whether or not a target had been present by pressing the left or right arrow key on the keyboard with the index or middle finger of the right hand, respectively.

In the VWM task, there was a blank interval of 2 seconds after stimulus onset, after which the memory probe (a single colored line in either the same or a different location as in the memory array) was presented for 160 ms. In the low memory load condition, participants were informed that the memory probe always had the same color (encouraging participants to only remember the location of a single line), whereas in the high memory load condition participants knew that the probe could have any of the seven colors (encouraging participants to remember all line locations). Participants made speeded responses within a 2-second response window after memory probe onset, indicating whether or not its color and location matched those of the memory array by pressing the left or right arrow key, respectively.

### Data preprocessing

Data from two participants in Experiment 1 and one participant from Experiment 2 were excluded from further analysis because no reliable pupil recordings could be obtained and one of the tasks could not be performed above chance level. In all experiments, trials were excluded when participants fixated anywhere more than 2.8° away from central fixation at any point in time between stimulus onset (memory array or search array) and response, as well as if any blink occurred within a 300-ms window around stimulus onset (on average, a total of 15% of trials in Experiment 1 and 9.5% of trials in Experiment 2). Pupil size (diameter change, in mm) was analyzed relative to a baseline period of 100 ms prior to onset of the first stimulus array for correct trials only. Blinks were reconstructed using cubic spline interpolation (Mathôt, 2013), and a five-point median Hampel filter was applied to remove artifacts.

### Online modeling of pupil dilation

To test if online modeling of pupil dilation can reliably indicate the current level of load or demand, as well as stimulus onsets, we employed an algorithm for online analysis of cognitive load from pupil data (based on previous work by Gollan & Ferscha, 2016). For all experiments, a static baseline was assumed (low enough to never exceed pupil dilation values) and subtracted from the measured pupil dilation levels while modeling the remaining pupil dilation levels as associated with cognitive loads and demands. Choosing a static baseline was justified by the current design of the study (where background luminance remained the same within a block of trials) and the absence of interactions between background luminance (gray vs. black) and behavioral performance or event-related (stimulus-locked and baselined) pupil responses (Note, though, that the modeled data were *not* time locked to stimulus onsets; instead, the algorithm worked on a continuous track and derived modeled pulses of pupillary responses through the measured pupil data itself.) However, implementing a dynamic baseline to compensate for environmental changes in luminance may improve the current algorithm in future iterations.

As a next step, the difference between static baseline and pupil diameter was modeled using an approach based on the task-evoked pupillary response (TEPR) (Hoeks & Levelt, 1993). This empirical model of pupillary response to cognitive activities has been transferred into an online analysis algorithm (Gollan & Ferscha, 2016). The online deconvolution algorithm performs a curve matching approach in a frame-wise feedback loop. The algorithm measures the difference between the current modeled pupil value and the current actual pupil dilation measure. If this difference exceeds a threshold of 0.25% change in pupil dilation, a new attentional pulse, *w_i_*(*s_i_,* *t_i_*), with scale *s_i_* and temporal onset *t_i_*, is dynamically added to the list of attentional impulses, *L*{*w_i_*}, at a temporal offset of *t* – 500 ms to the current detection time stamp and is optimized regarding scale. This iterative, dynamic curve-matching process efficiently performs online deconvolution of pupil dilation, providing both a modeled pupil dilation curve as well as the likely time stamps of the associated stimulus onsets.

The predicted pupil dilation pattern is derived from the online iterative matching process and can be reproduced offline by convolving the computed attention impulses:

$$M\;\left[t\right]=\sum_{i=1}^{j}\left(w_{i}*h\right)\left[t\right]\;=\sum_{i=1}^{j}s_{i}\;h\left[k_{i}-t\right]\;$$

with the pupillary light impulse response, *h*[*t*], empirically determined by Hoeks and Levelt (1993):

$$h\;\left[t\right]=t^{10.1}\;e^{\frac{-10.1\;t}{0.93}}$$

The online curve matching process is computed via the minimization of the error function between the modeled pupil dilation curve, *M*[*t*], and the observed actual pupil dilation measure, *Z*[*t*], as a least-squares problem, ε, optimizing the scale of the generated impulses to find the best possible match between modeled and observed pupil dilation levels:

$$ɛ\left(t_{i},s\right)\;=\sum_{T_{k}<t_{m}<t_{n}}{\left(M_{k}\left[t_{m},s\right]-Z\left[t_{m}\right]\right)}^{2}\;$$

A detailed description of the underlying optimization algorithm can be found in Gollan and Ferscha (2016).

### Analysis of original and modeled pupil dilation

To examine differences in pupil traces, linear mixed-effects (LME) analyses were used, with a criterion of *t* > 2 for significant effects, which is comparable to *p* < 0.05 (Baayen, Davidson, & Bates, 2008). LME models of the form *pupil size* ∼ *task* + (1 + *task*|*participant*), where pupil size is the dependent variable and task (e.g., high/low VWM or VS load) is the predictor variable with by-participant random intercept and slope, were constructed for each 16.67-ms pupil trace sample. (Note that, for analysis of pupil data with the continuous algorithm, the data were first modeled and only in a second step segmented for statistical and graphical comparison of pulse-locked and stimulus-locked pupil responses.) LME models to analyze the interaction in Experiment 1 included two predictor variables, VWM load and VS load, as well as by-participant random intercepts and slopes for each. Only sequences of at least 200 ms for which *t* > 2 were considered significant (Mathôt, Van der Linden, Grainger, & Vitu, 2013). Due to recent concerns about *p* value estimation for LME models, explicit *p* values are not reported, except for interactions (Blom, Mathôt, Olivers, & Van der Stigchel, 2016).

Because the results from the LME analyses provided evidence for distinct pupil responses to changes in memory loads and search demands across participants, we subsequently used classification by logistic regression to test trial-by-trial prediction of the level of load or demand in each of the two tasks. Predictive features of pupil traces were maximum pupil constrictions and dilations (as well as their latencies) within fixed time intervals where LME analysis had shown significant overall effects of memory load or search demand. Receiver operating characteristics (ROC) curves were constructed for each individual using the probabilities from the logistic regression model, distinguishing between high or low load or demand in each task based on one or more of the relevant features. The area under the curve (AUC) of the ROC provides a measure of trial-by-trial predictability for each participant, where an AUC of 0.5 is equal to chance classification performance (i.e., 50% correctly classified). Additionally, classification performance was estimated by fivefold cross-validation. For this, the data from each participant were partitioned into five sets. For each partition (the test set), a model was trained on all trials outside the partition (the training set), and then predictive performance was assessed on the trials in the test set itself. Classification accuracy was calculated as the average correct classification over all test sets. AUC was only considered if classification accuracy was significantly above chance.

To test the validity of the algorithm for load/demand discrimination, we analyzed individual modeled pupil traces in the same manner as the raw pupil data. (Note that one decisive difference was, of course, that the modeled data were *not* time-locked to the known stimulus onsets before impulses were derived.) This analysis of data from both experiments included LME analysis to test for differences between conditions and classification by logistic regression to assess how well specific features of the modeled pupillary response predict the level of load or demands on a single trial basis. Additionally, we examined the attentional pulses generated by the model to assess their temporal accuracy (i.e., their correspondence to the known stimulus onsets).

## Experiment 1. Combined VS and VWM load

### Behavioral data

**Figure 3.**
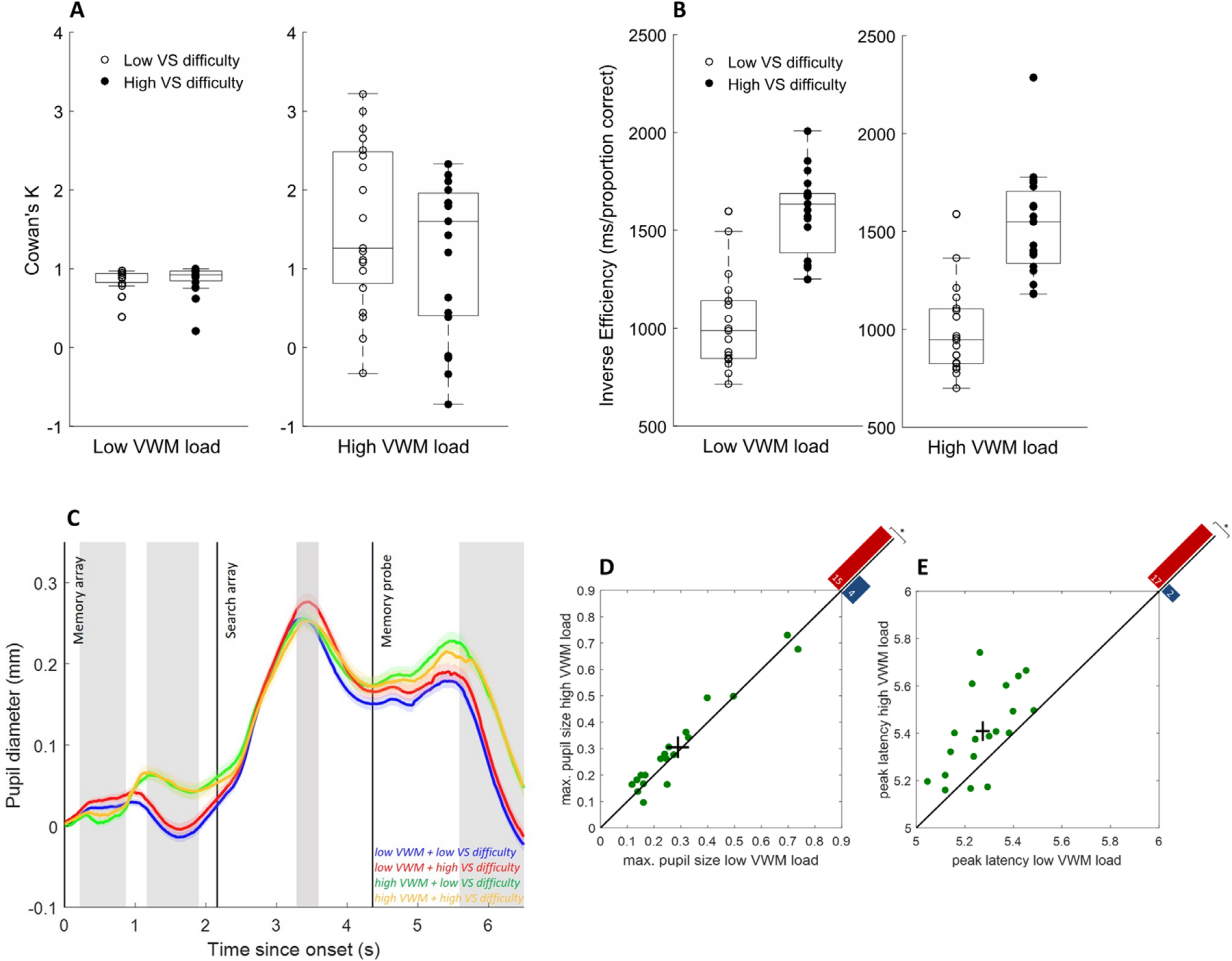
Behavioral performance and pupil traces in Experiment 1. (A) Cowan's *K* for the VWM task, indicating higher memory load when four items were kept in memory (high load) compared to one item (low load). (B) Inverse efficiency scores (RT/proportion correct) in the VS task, showing increased efficiency (lower values) under low compared to high VS difficulty, largely unaffected by concurrent VWM load. In each box, the central horizontal line is the median; the edges of the box are the 25th and 75th percentiles. Data points represent individual participants. (C) Change in pupil diameter as a function of memory load and search difficulty. Light-gray shading represents periods of significant memory load effects (at least 200 ms of consecutive samples where *t* > 2). Gray shading at the center indicates a period of significant interaction between memory load and search difficulty. Dark, vertical lines indicate stimulus onsets. Error shading is ±1 *SE* pooled across all trials (not weighted by participant). Zero on the *y*-axis indicates mean baseline pupil size. (D) A significant fraction of individuals (each dot corresponds to one participant) showed greater peak pupil size and reached peak pupil size at a later point in time (E) after memory probe onset in the high (*y*-axis) compared to low (*x*-axis) VWM load condition. The black cross denotes average over participants and 1 *SE* in each dimension. Red and blue bars in top right corners depict the number of points above and below the diagonal (significant sign-test).

Repeated measures ANOVAs, with independent variables of memory load and search difficulty, were conducted on mean RTs for correct trials and accuracy data (proportion correct). Trials where RTs were shorter than 200 ms or exceeded 2500 ms were excluded from further analysis (3.14% on average). For the most important significant results, see Figures 3A and 3B.

In the VS task, participants responded significantly faster in the low-difficulty condition (*M* = 863 ms) compared to the high-difficulty condition (*M* = 978 ms), where *F*(1, 18) = 11.82, *p* < 0.01, and ηp² = 0.40. Moreover, memory load significantly reduced RTs in the search task. Irrespective of the difficulty of the search task, participants responded on average 34 ms faster in the VS task when concurrent memory load was high (*M* = 904 ms) compared to when memory load was low (*M* = 938 ms), where *F*(1, 18) = 9.62, *p* < 0.01, and ηp² = 0.35. Participants also performed more accurately in the search task under low search difficulty (*M* = 87%) compared to high search difficulty (*M* = 63%), where *F*(1, 18) = 245.35, *p* < 0.001, and ηp² = 0.93. However, concurrent memory load had no effect on accuracy in the VS task (*p* = 0.94). Furthermore, there were no interactions between memory load and search difficulty for RTs or accuracy (all *p* > 0.7).

To check for speed–accuracy tradeoffs, inverse efficiency (RT/proportion correct) scores were computed (Townsend & Ashby, 1983). Analysis of inverse efficiency scores showed better performance in the VS task when search difficulty was low (*M* = 1009 ms/proportion correct) compared to when it was high (*M* = 1558 ms/proportion correct), where *F*(1, 18) = 98.43, *p* < 0.001, and ηp² = 0.85, as well as a marginal effect of concurrent memory load, where *F*(1, 18) = 4.29, *p* = 0.053, and ηp² = 0.19, with performance being slightly better under high memory load (*M* = 1260 ms/proportion correct) compared to low memory load (*M* = 1307 ms/proportion correct). There was no interaction for inverse efficiency between search difficulty and memory load in the VS task (*p* > 0.7). The behavioral results from the VS task confirm that the search difficulty manipulation was effective. Increasing memory load, however, had no detrimental effect on search performance; thus, additive factors logic would suggest that the involved resources occupied by the VWM versus the VS task were independent from one another (cf. Sternberg, 1969).

In the VWM task, participants responded significantly faster and more accurately under low memory load (*M* = 658 ms; *M* = 93%) compared to high memory load (*M* = 975 ms; *M* = 67%), where *F*(1, 18) = 164.33, *p* < 0.001, ηp² = 0.90 and *F*(1, 18) = 104.34, *p* < 0.001, ηp² = 0.86, for RTs and accuracy, respectively. Search difficulty had no effect on RTs or accuracy in the VWM task (all *p* > 0.10). However, there was a marginally significant interaction between memory load and search difficulty on accuracy in the VWM task, where *F*(1, 18) = 3.96, *p* = 0.062, and ηp² = 0.18. Analysis of inverse efficiency scores in the VWM task showed a significant effect of memory load only, where *F*(1, 18) = 110.40, *p* < 0.001, and ηp² = 0.86. There were no other significant effects or interactions (all *p* > 0.24). To assess the effectiveness of the load manipulation in the VWM task, VWM estimates were calculated: Cowan's *K* = *N* × (hit rate – false alarm rate), where *K* is the VWM estimate and *N* is the number of items in the memory array, here one vs. four (Cowan, Elliott, Saults, Morey, Mattox, & Hismjatullina, 2005). A repeated measures ANOVA on the VWM estimates revealed a significant increase with high memory load (*K* = 1.37, *SE* = 0.23) compared to low memory load (*K* = 0.87, *SE* = 0.04), where *F*(1, 18) = 6.00, *p* < 0.05, and ηp² = 0.25, confirming that the memory load manipulation was effective and that the high memory load condition occupied more VWM capacity than the low memory load condition. Furthermore, the analysis revealed a marginally significant interaction between VS difficulty and memory load for the VWM estimates, where *F*(1, 18) = 4.32, *p* = 0.052, and ηp² = 0.19, suggesting a smaller memory load effect under high VS difficulty compared to low VS difficulty. Importantly, additional ANOVAs showed that background luminance had no effect on task performance (RTs and accuracy) in both tasks (all *p* > 0.08).

### Pupil traces

For a conventional analysis of the eye data, the continuous pupil data were segmented into 6.5-second epochs time-locked to the memory array onset and baselined. (Note that this analysis is *not* to be confused with application of the algorithm. As explained, the algorithm was applied online, in a continuous fashion, and impulses corresponding to the onsets of the tasks were automatically derived.) Separate LMEs were conducted for interactions between background luminance and search difficulty or memory load. To test for differences in each condition, additional LMEs were conducted for search difficulty and memory load and their interactions as fixed effects. All LME models were constructed with by-participant random intercepts and slopes for all fixed effects, with pupil surface (diameter change from baseline in mm) as the dependent measure, and they were conducted for each 16.67-ms sample separately.

Although pupil traces showed large absolute differences between light background conditions (small pupil diameter) and dark background conditions (large pupil diameter), after baseline subtractions LME analysis of pupil data showed no significant effects of background luminance in either experiment (i.e., no sequence of at least 200 ms where *t* > 2 or *p* < 0.05 for interactions). This suggests that background luminance did not modulate search difficulty or memory load influences on pupil responses. Clear differences emerged between the pupil responses under low and high VWM load after presentation of the memory array (where items were encoded and maintained in memory) as well as after presentation of the memory probe (where the probe was matched to the items in the initial memory array and a response was required). Generally, pupil size increased under higher memory loads during encoding, as well as during recognition, irrespective of the difficulty level of the intermittent search task (Figure 3C). However, during the encoding stage, this pattern was initially reversed, showing slightly larger pupil sizes under low memory demands. This slight initial constriction under high memory load may be due to the increased pupillary light reflex elicited by the larger stimulus set sizes (four vs. one item in the high compared to low VWM condition) (cf. Barbur, Harlow, & Sahraie, 1992; Kohn & Clynes, 1969). Experiment 2 controlled for these effects by using memory arrays with an equal number of items in different load conditions.

To identify characteristics of the pupil traces that consistently indicated the level of load in the VWM task, mean maximum dilations and constrictions from each individual were compared within the critical time windows identified in the LME analysis. Maximum constriction in the first two time windows after memory array onset (300–900 ms and 1.2–1.9 seconds) consistently indicated the level of VWM load for 17 and 11 out of 19 participants, where *t*(18) = 3.07, *p* < 0.01 and *t*(18) = 2.19, *p* < 0.05, respectively, for paired *t*-tests. Furthermore, for a significant fraction of participants, the maximum dilation and its time point (peak latency) after memory probe onset indicated the level of VWM load (15/19 and 17/19 participants; *p* < 0.05, sign-tests) (Figures 3D and 3E). When effect sizes were considered, however, significance only prevailed for peak latency, where *t*(18) = 4.11 and *p* < 0.001, paired *t*-test.

Effects of VS difficulty were generally less pronounced and were absent in the immediate pupil response to the memory array and memory probe; however, around the period of maximum pupil dilation in each trial, LME analysis revealed a significant VS difficulty and VWM load interaction (1123–1440 ms after search array onset). During this period, the change in pupil size was larger under high compared to low VS difficulty, but this was only the case when concurrent memory load was low. When memory loads were high, search demands had no effect on pupil size. This suggests that any pupil size modulation due to increased VS difficulty may have been suppressed under high memory load.

To identify characteristic features that indicated the level of load in the search task, maximum dilations from each individual (and their latencies) were compared within the time window from search array onset to memory probe onset. Although maximum dilation (as well as mean pupil size) during the search interval (from search array onset to memory probe onset) was on average not indicative of search load (*p* > 0.10), the latency of maximum dilation did significantly differ between the conditions, where *t*(18) = 4.13 and *p* < 0.01.

The results suggest a characteristic time course of pupillary responses depending on the level of load or difficulty in each of the two tasks, respectively. Maximum dilations and their latencies within predefined time windows indicated the level of load in each task for a significant fraction of participants, as well as on average. To test if specific features of the response can also predict the level of search difficulty or memory load from a single pupil trace on a given trial, we classified individual pupil traces using logistic regression. Based on the results of the LME analyses, we identified several pupil response features to include in the classification of single trial pupil data: maximum constriction from 1.2 seconds after memory array onset (the point at which the traces for low and high memory load diverged) until search array onset, maximum dilation from search array onset until memory probe onset, and maximum dilation after memory probe onset. In addition, the time points (latencies) of each maximum were used as additional features. For binary classification, data were analyzed separately for the search and memory tasks. VS difficulty classification accuracy (after fivefold cross-validation) was strongest when all features were included in the analysis (*M* = 59.8%, *SE* = 1.56). Classification accuracy, as well as the corresponding AUC (*M* = 0.66, *SE* = 0.016), were on average significantly above chance: *t*(18) = 6.29, *p* < 0.0001 and *t*(18) = 10.35, *p* < 0.0001, respectively. The strongest single feature to predict current search difficulty significantly above chance was, as expected, the magnitude of maximum dilation between search array and memory probe onset: for classification accuracy, *M* = 60.1%, *SE* = 0.84, *t*(18) = 12.07, and *p* < 0.0001; for AUC, *M* = 0.57, *SE* = 0.014, *t*(18) = 5.08, and *p* < 0.001.

For the memory task, classification for level of load was again strongest when all features were included in the analysis: for classification accuracy, *M* = 67.9%, *SE* = 1.58, *t*(18) = 11.37, and *p* < 0.0001; for AUC, *M* = 0.73, *SE* = 0.019, *t*(18) = 12.05, and *p* < 0.0001. However, the strongest single feature that could still predict memory load significantly above chance was the latency of maximum dilation after memory probe onset: for classification accuracy, *M* = 63.2%, *SE* = 2.15, *t*(18) = 6.12, and *p* < 0.0001; for AUC, *M* = 0.59, *SE* = 0.016, *t*(18) = 5.96, and *p* < 0.0001.

### Analysis of modeled pupil response

**Figure 4.**
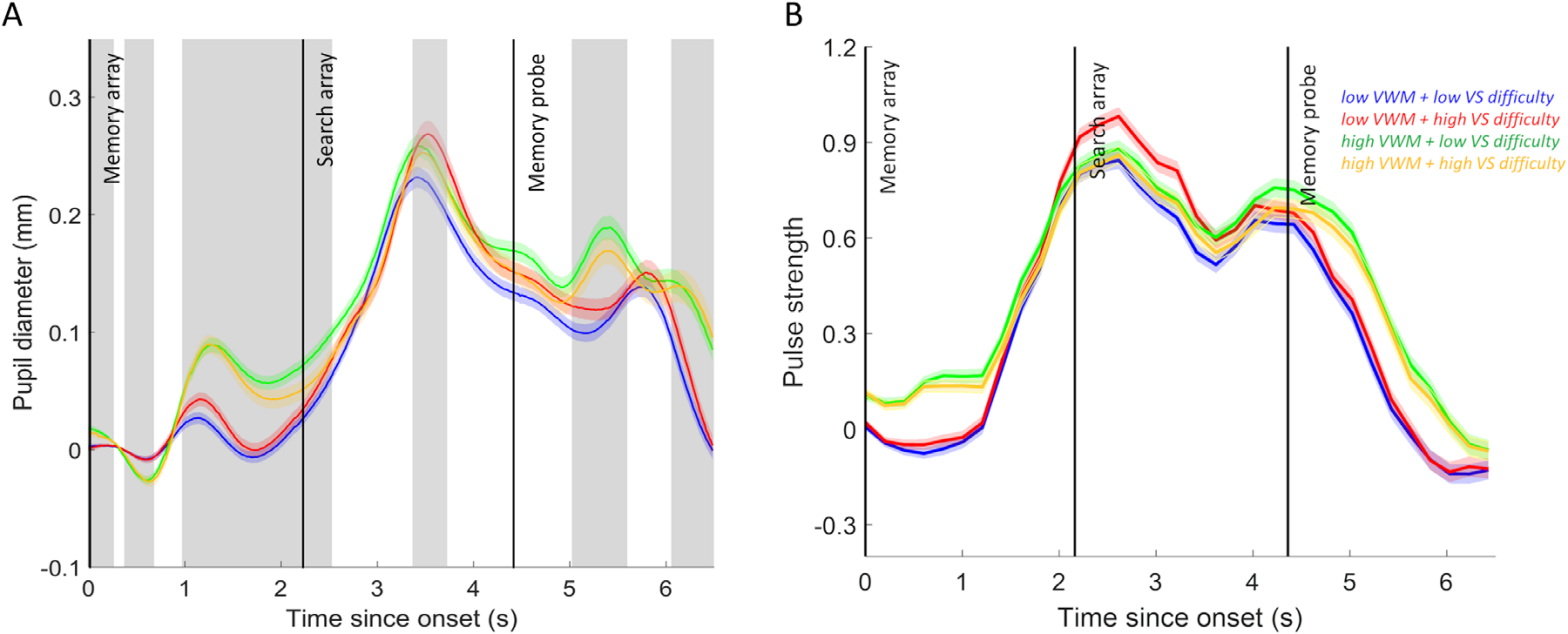
(A) Change in pupil diameter of online modeled continuous pupil traces as a function of memory load and search difficulty. Gray shading represents periods of significant memory load (VWM) effects (at least 200 ms of consecutive samples where *t* > 2) or search difficulty (VS) effects (shading in center of figure). (B) Average strength of attentional pulses per condition. Peak attentional pulses appear to roughly indicate stimulus onsets, especially for the memory probe. Dark, vertical lines indicate stimulus onsets. Error shading is ±1 *SE* pooled across all trials. Zero on *y*-axis indicates mean baseline pupil size (or pulse strength).

To test the ability of the algorithm to indicate the level of VS difficulty, we analyzed individual modeled pupil traces in the same manner as the stimulus-locked pupil data. The continuous, modeled data were cut into 6.5-second epochs (from memory array onset) and baselined. We used LME analysis on each sample with a criterion of *t* > 2 for significant effects in order to identify periods where conditions differed significantly for consecutive samples of at least 200 ms (Figure 4A).

To compare single trial classification performance of the modeled and stimulus-locked pupil data, we repeated the classification analysis with the same features as before. To allow binary classification, data were again analyzed separately for the search difficulty and memory load tasks. VS difficulty classification (after fivefold cross-validation) was successful when all features were included in the analysis (*M* = 57.8%, *SE* = 1.30). Classification accuracy and the corresponding AUC (*M* = 0.65, *SE* = 0.013) were on average significantly above chance: *t*(18) = 5.97, *p* < 0.0001 and *t*(18) = 11.76, *p* < 0.0001, respectively. The strongest single feature to predict current search difficulty significantly above chance was, as for the stimulus-locked pupil data, the magnitude of maximum dilation between search array and memory probe onset: for classification accuracy, *M* = 60.0%, *SE* = 1.11, *t*(18) = 9.02, and *p* < .0001; for AUC, *M* = 0.57, *SE* = 0.011, *t*(18) = 6.16, and *p* < .0001.

In the memory task, classification for level of load in the modeled data was again strongest when all features were included in the analysis: for classification accuracy, *M* = 66.0%, *SE* = 1.70, *t*(18) = 9.36, and *p* < 0.0001; for AUC, *M* = 0.71, *SE* = 0.016, *t*(18) = 13.34, and *p* < 0.0001. However, the strongest single feature that could still predict memory load significantly above chance was the latency of maximum dilation after memory probe onset: for classification accuracy, *M* = 63.7%, *SE* = 1.89, *t*(18) = 7.26, and *p* < 0.0001; for AUC, *M* = 0.57, *SE* = 0.013, *t*(18) = 5.33, and *p* < 0.0001.

### Attentional pulses

To test if modeled attentional pulse strength predicted stimulus onsets, we averaged the strongest modeled pulses within each condition for periods of ±1 second around each stimulus onset (memory array onset, search array onset, and memory probe onset). Under high memory load, maximum pulse timing did not significantly differ from actual memory array onset (Table 1). Thus, pulse strength in this condition correctly reflected memory array onset. Under low memory load, the predicted onsets were slightly prior to actual onset (∼ –50 ms). The same pattern was found for memory probe onsets. Here, pulse strength correctly reflected stimulus onset under high memory load and again predicted onsets slightly before actual onsets (∼ –57 ms) when memory load was low. For the search array onset, modeled pulses were on average strongest ∼143 ms after actual onset.

**Table 1.** Time points of strongest deconvolved attentional pulses relative to stimulus onsets in each condition of Experiment 1 (CIs and *p* values of one-sample *t*-tests) *Notes:* LM = low memory load; HM = high memory load; LS = low search load; HS = high search load.

Onset	Condition	Time of maximum pulse (to onset; ms)	CI	*p*
Memory array	LM/LS	–53	−83, –24	<0.01
	LM/HS	–46	–83, –9	<0.05
	HM/LS	14	–37, 47	0.81
	HM/HS	5	–28, 55	0.51
Search array	LM/LS	141	88, 19	<0.001
	LM/HS	163	123, 202	<0.001
	HM/LS	119	76, 162	<0.001
	HM/HS	147	102, 193	<0.001
Memory probe	LM/LS	–55	–91, –19	<0.01
	LM/HS	–59	–99, –19	<0.01
	HM/LS	1	–43, 42	0.98
	HM/HS	6	–40, 51	0.79

## Experiment 2. Separate VS and VWM load

### Behavioral data

#### Visual search task

Repeated measures ANOVAs, with independent variables search type (more vs. less efficient), set size (two vs. seven), and background luminance (dark vs. light), were conducted on mean RTs for correct trials and accuracy data (proportion correct). Trials where RTs were shorter than 200 ms or exceeded 2500 ms were excluded from further analysis (1% on average). Performance was better when searching for a tilted line among vertical lines (efficient search) compared to searching for a vertical line among tilted lines (less efficient search). Participants responded on average significantly faster in the more efficient search condition (*M* = 535 ms) compared to the less efficient search condition (*M* = 577 ms), where *F*(1, 15) = 20.67, *p* < 0.001, and ηp² = 0.58. Moreover, a significant interaction between search type and set size for RTs validated the experimental manipulation by demonstrating slower RTs due to the increased set size only in the less efficient search condition and not in the more efficient search condition, where *F*(1, 15) = 9.89, *p* < 0.01, and ηp² = 0.40.

**Figure 5.**
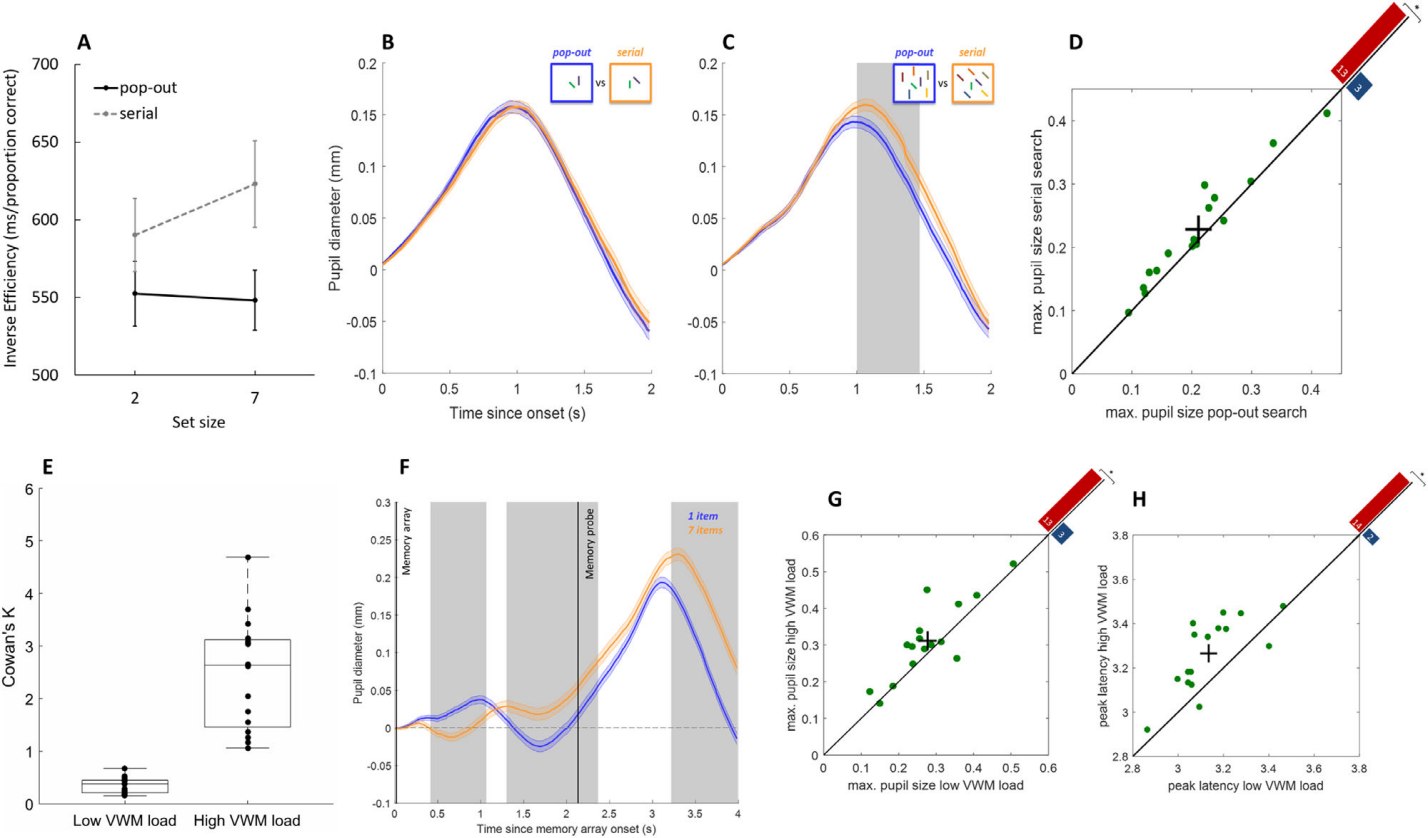
Behavioral performance and pupil traces in Experiment 2. A. Inverse efficiency scores (RT/proportion correct) in the VS task, showing increased efficiency (lower values) in the more efficient (pop-out) search condition and decreased efficiency with increasing set size in the less efficient search condition. Error bars indicate ±1 *SE*. Change in pupil size as a function of set size, either two items (B) or seven items (C), and search type (blue, more efficient pop-out search; yellow, less efficient serial search). Observers detected the presence of a tilted target (more efficient search) or vertical target (less efficient search) in each condition (here, small green bar in insets). Differences in pupil response only emerged when set size was large (C). Gray shading represents period of significant search-type effect (at least 200 ms of consecutive samples where *t* > 2). Error shading indicates ±1 *SE*. (D) A significant fraction of individuals (one per dot) showed greater peak pupil sizes during less efficient search (*y*-axis) compared to more efficient search (*x*-axis) in the large set size condition. The black cross denotes average over participants and 1 *SE* in each dimension. Red and blue bars in top right corner denote the number of points above and below the diagonal (significant sign-test). (E) Cowan's *K* for the VWM task, indicating higher memory load when seven items were kept in memory compared to one item. In each box, the central horizontal line is the median; the edges of the box are the 25th and 75th percentiles. Data points represent individual participants. (F) Change in pupil size as a function of VWM load. Gray shading represents periods of significant VWM load effect. A significant fraction of individuals (each dot corresponds to one participant) showed greater peak pupil sizes (G), as well as longer peak latencies (H), during high VWM load (*y*-axis) compared to low VWM load (*x*-axis).

To check for speed–accuracy tradeoffs, inverse efficiency (RT/proportion correct) scores were computed. Analysis of inverse efficiency scores mirrored the results from the RT analysis. Performance was better in the more efficient search condition (*M* = 550 ms/proportion correct) compared to the less efficient search condition (*M* = 607 ms/proportion correct), where *F*(1, 15) = 28.74, *p* < 0.001, and ηp² = 0.66. Also, there was a significant interaction between search type and set size, where *F*(1, 15) = 7.91, *p* < 0.05, and ηp² = 0.35, indicating reduced efficiency in the more difficult search condition when set size increases (see Figure 5A). Importantly, again, background luminance had no significant effects on task performance (all *p* > 0.2).

#### Memory task

Repeated measures ANOVAs, with independent variables memory load (one vs. seven items) and background luminance (dark vs. light), were conducted on mean RTs for correct trials and accuracy data (proportion correct). Trials where RTs were shorter than 200 ms or exceeded 2500 ms were excluded from further analysis (<1% on average). Performance in the memory task was significantly better in the low memory load condition (RT, *M* = 580 ms; percent correct, *M* = 92.76%) compared to the high memory load condition (RT, *M* = 862 ms; percent correct, *M* = 68.74%): for RTs, *F*(1, 11) = 120.62, *p* < 0.001, and ηp² = 0.92; for accuracy, *F*(1, 11) = 102.34, *p* < 0.001, and ηp² = 0.90. To further assess the effectiveness of the VWM task, VWM estimates were calculated (Cowan's *K*, as in Experiment 1). A repeated measures ANOVA on the VWM estimates revealed a significant increase with high memory load (*K*, *M* = 2.62; *SE* = 0.31) compared to low memory load (*K*, *M* = 0.37; *SE* = 0.05), where *t*(11) = 8.35 and *p* < 0.001, confirming that the memory load manipulation was effective and that the high load condition occupied more VWM capacity than the low load condition (Figure 5E). Again, background luminance had no effect on performance in the VWM task (all *p* > 0.4).

### Pupil traces

#### Visual search task

As in Experiment 1, separate LMEs were conducted for interactions between background luminance and set size (one vs. seven) and background luminance and search type (more efficient vs. less efficient), as well as the interaction between set size and search type in conventionally analyzed eye-tracking data. To test for effects of search type (more efficient vs. less efficient), additional LMEs were conducted for search type as fixed effect in each set size condition. As was the case previously, all LME models used by-participant random intercepts and slopes for all fixed effects (and their interactions), used change in pupil diameter as a dependent measure, and were conducted for each 16.67-ms sample separately.

After baseline subtraction, there were neither significant effects of background luminance nor interactions between background luminance and search type or background luminance and set size (i.e., no sequence of at least 200 ms where *t* > 2 or *p* < 0.05 for interactions). The interaction between search type and set size was significant around the period of maximum pupil dilation (683–1483 ms after search array onset). To test for differences between the search type conditions depending on set size, separate LMEs were conducted for search type as fixed effect when set size was either two or seven. Based on the behavioral results (Figure 5A), we expected greater pupil modulation for less efficient compared to more efficient search, but only with the large set size and no effect with the small set size. The results confirmed this prediction, demonstrating significantly larger pupil dilation for less efficient search compared to more efficient search (1 second to 1467 ms after search array onset) only in the large set size condition (Figure 5C).

Maximum dilation after search array onset was a consistent feature of the pupil response indicating the type of VS in 13 out of 16 participants (Figure 5D). This fraction was significant even when effect sizes in individuals were ignored (*p* = 0.02, sign test). When effect sizes were considered and mean peak dilations for more and less efficient searches were compared, significance prevailed: *t*(15) = 2.96 and *p* < 0.01, paired *t*-test. Thus, for a significant majority of individuals, and on average, peak pupil dilation in response to the large set VS array was greater for inefficient (serial) search compared to efficient (pop-out) search.

Individual pupil traces were classified using logistic regression to test if the differences in response to search type observed at the group level could be identified from specific features of single trial pupil traces. Accuracy of classification of VS difficulty (after fivefold cross-validation) was significantly above chance when maximum dilation and the time point of maximum dilation after stimulus onset were both included as predictors in the analysis: for classification accuracy, *M* = 55.0%, *SE* = 1.58, *t*(15) = 2.91, and *p* < 0.05; for AUC, *M* = 0.60, *SE* = 0.012, *t*(15) = 7.47, and *p* < 0.0001. The only significant single predictor was latency of maximum dilation: for classification accuracy, *M* = 55.0%, *SE* = 1.48, *t*(15) = 3.10, and *p* < 0.01; for AUC, *M* = 0.58, *SE* = 0.016, *t*(15) = 5.55, and *p* < 0.001.

#### Memory task

Similarly to the search task, in the memory task results from the LME analyses demonstrated neither a significant effect of background luminance nor an interaction between background luminance and memory load (i.e., no sequence of at least 200 ms where *t* > 2 or *p* < 0.05, for interactions) after baseline subtraction. However, there were three intervals of significant memory load effects (at 417–1067 ms, 1300–2367 ms, and 3217 ms to 4 seconds from memory array onset) (Figure 5F). Although pupil responses were generally larger under high compared to low memory load, this was not the case for the first interval (417–1067 ms after onset) where increased memory load initially resulted in stronger constriction. This pattern was similar to Experiment 1 (see Figure 3C) despite the fact that the memory task in Experiment 2 always contained the same number of items in the encoding array.

Maximum pupil dilation and its latency consistently predicted the level of VWM load in 13 out of 16 and 14 out of 16 participants, respectively (Figures 5G and 5H). These fractions were significant with or without consideration of effect sizes (*p* < 0.05, sign-tests): *t*(15) = 2.43, *p* < 0.05 and *t*(15) = 4.40, *p* < 0.001 for maxima and latencies at maxima, respectively.

To predict the level of memory load from individual pupil traces, we chose features similar to those in Experiment 1 based on the results from LME analyses (i.e., maximum constrictions and dilations in the three intervals where significant differences were observed across participants, as well as their respective latencies) to include in the classification model. Mean classification accuracy (*M* = 66.4%, *SE* = 1.49) and AUC (*M* = 0.76, *SE* = 0.015) were significantly above chance when all predictors were included in the logistic regression model: *t*(15) = 10.13, *p* < 0.0001 and *t*(15) = 15.62, *p* < 0.0001, respectively. The strongest single predictor to successfully classify memory load in a given trial was the latency of the maximum pupil dilation after memory probe onset: for classification accuracy, *M* = 62.3%, *SE* = 1.26, *t*(15) = 8.96, and *p* < 0.0001; for AUC, *M* = 0.64, *SE* = 0.021, *t*(15) = 5.92, and *p* < 0.0001.

### Analysis of modeled pupil response

#### Visual search task

**Figure 6.**
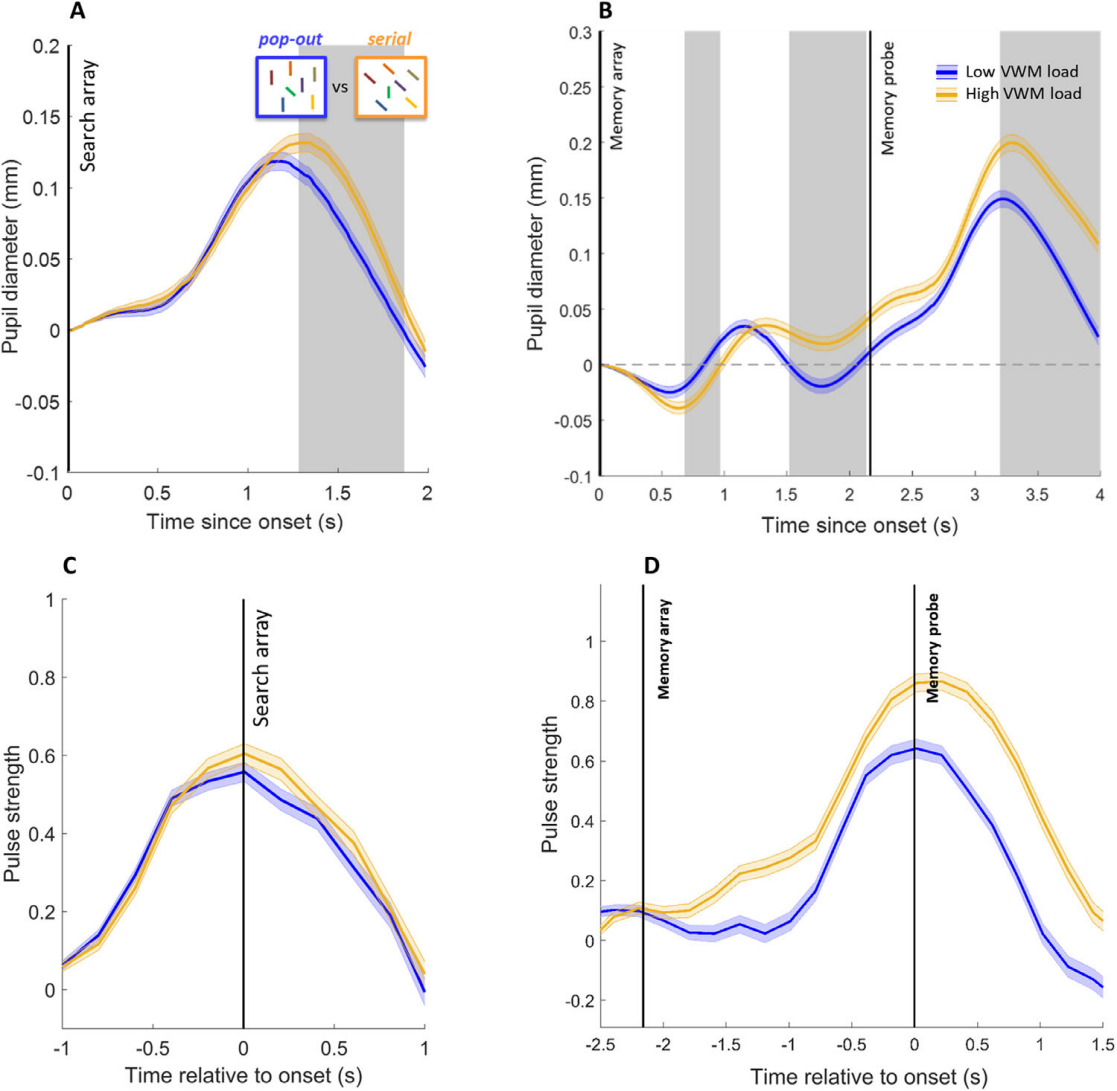
(A) Change in pupil size of online modeled continuous pupil traces as a function of search type (blue, more efficient search; yellow, less efficient search) in the large set size condition only. (The low set size condition is not depicted.) As in the stimulus-locked data, differences in pupil response only emerged when set size was large. These differences were more pronounced and were significant for a longer period compared to the stimulus-locked data (Figure 5C). Gray shading represents period of significant search type effect (at least 200 ms of consecutive samples where *t* > 2). (B) Change in pupil size of modeled data as a function of VWM load (one or seven memory items). (C) Average strength of attentional pulses per condition in the VS task. Modeled peak attentional pulses correspond well to stimulus onsets in both conditions. (D) Average strength of attentional pulses per condition in the VWM task. Modeled peak attentional pulses roughly correspond to stimulus onsets (slightly more so in the low load conditions compared to the high load conditions). Error shading indicates ±1 *SE* pooled across all trials.

We employed the same algorithm for online analysis of memory load and search difficulty from pupil data of Experiment 2. To test the ability of the algorithm to indicate the level of VS difficulty, we analyzed individual modeled pupil traces in the same manner as the stimulus-locked pupil data. (These pupil traces were first calculated online without knowledge of the stimulus onsets, only with the help of modeled impulses.) The continuous modeled data were cut into 2-second epochs (starting at search array onset) and baselined. We then used LME analysis on each sample with a criterion of *t* > 2 for significant effects in order to identify periods where conditions differed significantly for consecutive samples of at least 200 ms (Figure 6A).

Accuracy of classification of VS difficulty (after fivefold cross-validation) was significantly above chance when maximum dilation and the time point of maximum dilation after stimulus onset were both included as predictors in the analysis: for classification accuracy, *M* = 53.8%, *SE* = 1.57, *t*(15) = 2.23, and *p* < 0.05; for AUC, *M* = 0.60, *SE* = 0.013, *t*(15) = 6.88, and *p* < 0.0001. The only significant single predictor was latency of maximum dilation: for classification accuracy, *M* = 54.3%, *SE* = 1.67, *t*(15) = 2.38, and *p* < 0.05; for AUC, *M* = 0.58, *SE* = 0.013, *t*(15) = 5.55, and *p* < 0.001.

#### Attentional pulses

In both search conditions, modeled maximum pulse timing did not differ significantly from actual search array onsets (Table 2); thus, pulse strength correctly reflected stimulus onset.

**Table 2.** Time points of strongest deconvolved attentional pulses relative to stimulus onsets in each condition of the search task in Experiment 2. (CIs and *p* values of one-sample *t*-tests).

Onset	Condition	Time of maximum pulse (to onset; ms)	CI	*p*
Search array	Efficient search	–16	−76, 37	0.47
	Inefficient search	0	−56, 55	0.99

#### Memory task

We tested again whether or not the modeled pupil data produced similar results and reliably indicated the level of memory load in the task. The results (Figure 6B) showed a similar pupil response as in the stimulus-locked data (Figure 5F), reflecting significant differences in VWM load at roughly the same time points.

Mean classification accuracy (*M* = 65.5%, *SE* = 1.15) and AUC (*M* = 0.75, *SE* = 0.015) were significantly above chance when all predictors were included in the logistic regression model: *t*(15) = 12.35, *p* < 0.0001 and *t*(15) = 14.68, *p* < 0.0001, respectively. The strongest single predictor to successfully classify memory load in a given trial was the latency of the maximum pupil dilation after memory probe onset: for classification accuracy, *M* = 61.0%, *SE* = 1.33, *t*(15) = 7.60, and *p* < 0.0001; for AUC, *M* = 0.63, *SE* = 0.020, *t*(15) = 5.99 and *p* < 0.0001.

#### Attentional pulses

Similar to the modeled data from Experiment 1, maximum attentional pulse strength in the memory task of Experiment 2 occurred earlier in the low memory load condition compared to the high memory load condition. Under high memory load, stimulus onsets (memory array onset and memory probe onset) were predicted slightly after actual onsets (∼55 ms); whereas, under low memory load, predicted onsets did not differ significantly from actual onsets, so pulse strength correctly reflected stimulus onsets (Figure 6D and Table 3).

**Table 3.** Time points of strongest deconvolved attentional pulses relative to stimulus onsets in each condition of the memory task in Experiment 2. (CIs and *p* values of one-sample *t*-tests).

Onset	Condition	Time of maximum pulse (to onset; ms)	CI	*p*
Memory array	Low memory	–25	–68, 18	0.24
	High memory	50	9, 91	<0.05
Memory probe	Low memory	–12	–71, 47	0.68
	High memory	60	4, 116	<0.05

## Discussion

In the current study, we set out to validate a recently developed algorithm for the continuous online assessment of cognitive demands or task load from pupil dilation responses that could ultimately be used instead of other more performance-directed measures, such as speed of cognitive performance or degree of accuracy. Such an alternative measure would have the advantage of being usable in a variety of different cognitive work contexts, with covert performance and without (prior) knowledge of the exact time of task or processing onset. However, as pupil dilations are also influenced by a number of other factors, most notably luminance, it is not clear how well such an algorithm works. The current study, therefore, validated how such an algorithm fares in a laboratory experiment with two different tasks: a VWM task and a VS task.

In addition, the study of both accuracy and speed of task performance and of pupillary responses in these tasks is also interesting in its own right, as (1) we combined the two tasks within each trial of Experiment 1 to investigate if VWM and VS tasks interact, and (2) not much is known about pupillary responses under varying VS demands at all.

Concerning the speed and the accuracy of performance, results were as expected, with one exception. First, regarding VS task performance, as expected, increasing search difficulty from conditions with less target-distractor similarity to conditions with more target-distractor similarity (in Experiment 1) and from the easier to the more difficult version of a search asymmetry protocol (Experiment 2), as well as increasing the number of distractors only in the more difficult version of the search asymmetry protocol (Experiment 2), all led to a drop in VS performance (longer RTs and higher inverse efficiency scores). Second, also as expected, increasing the memory load prompted lower performance in Experiments 1 and 2 (longer RTs and reduced accuracy). The only unexpected result concerned a paradoxical facilitation of VS performance under the high memory load compared to the low-memory load conditions of Experiment 1. This was also the only indication of an interaction between VS and VWM task performance in the reaction times and accuracies. Possibly, at the start of a trial, during the VWM task participants strategically over-compensated for the anticipated VS task in trials with a high memory load.

Note that participants in Experiment 1 knew from the number of stimuli in the encoding (memory array) display if the memory load was low or high. If they decided to spare resources that are shared with the VS task for the VS task in the high memory load conditions, but not or less so in the low memory load condition, then it is possible that even more resources were available for VS performance in the high memory load conditions compared to the low memory load conditions. This, however, was the only indication of an interaction between the two tasks in the performance data. Importantly, the pattern of the interaction is completely different from the predictions of mutual interference between VWM and VS task. Thus, on the basis of the results, it is perfectly possible that there was no interference between VWM and VS task in the current study at all. A reason could be that participants could have searched for the targets in the VS task with the help of a long-term memory representation (e.g., Carlisle, Arita, Pardo, & Woodman, 2011) that interfered less with VWM content. However, it is also possible that VWM and VS do not draw on shared resources under all conditions (cf. Tas, Luck, & Hollingworth, 2016).

Whatever the reason, the pupillary responses supported an interpretation of little mutual interference between VS and VWM task. Although pupil responses validly reflected increased difficulties of the VS task and higher loads of the VWM tasks, results of Experiment 1 largely demonstrated independent effects of VS difficulty and VWM load manipulations on pupil size, despite the better performance in the VS task under high memory load VWM conditions. In fact, the single indication of an interaction between VS and VWM tasks over and above the interaction in the performance data that we found in the pupillary responses was a missing effect of search difficulty under high memory load but not under low memory load conditions at the time following the VS display onset of Experiment 1. If this interaction would have indeed reflected that VWM load and perceptual difficulty draw on a common resource, such that no further dilation by VS search difficulty could be observed, we would have expected to see a corresponding interaction in the overt performance measures. As this was not the case, we think that it is more likely that not all pupil dilation effects reflected demands or loads only. For example, it is possible that more negative evaluations of the more difficult VWM task under high memory load conditions dominated or masked the demand-elicited VS task difficulty differences, as negative evaluations can also lead to pupil dilations (e.g., Pool, Pauli, Kress, & O'Doherty, 2019). Thus, the current design does not allow drawing strong conclusions with regard to shared resources between VS and VWM tasks. Future studies should address this question with more suitable paradigms that reliably produce interactive effects between VS and VWM load in behavioral performance measures.

In any case, one should note that, by conducting the VS task in the retention interval of the VWM task, we sought to provide a challenging test of the pupillary response as a reflection of demands in general and of the algorithm in particular, as this task combination aimed to mimic the relatively complex meshing of different cognitive demands that is typical of more applied work contexts. This scenario carries some ecological validity but makes it clear that there are limits to the degree to which current task demands can be inferred on the basis of pupil sizes alone.

The results of Experiment 2 are consistent with those of Experiment 1. Again, task difficulty had the expected effects on VWM and VS task performance: Participants were slower in the more difficult version of the VS asymmetry protocol than in the easier one, and, only in the more difficult version, search times increased with increasing distractor number, indicative of inefficient search. In contrast, in the easier version, search times were independent of the number of distractors presented. In the VWM task, performance was better with a lower than with a higher memory load. In addition, the observed pupillary response time course for encoding into VWM, maintaining, and recognizing items from VWM replicated those in Experiment 1. The pupil dilation discriminated between the number of items held in memory, and responses were successfully classified accordingly on a trial-by-trial basis with relatively high sensitivity (AUC of 0.76 on average). In addition, results from the VS task demonstrated differences in pupillary response between more efficient (pop-out) search and less efficient search. These findings expanded the results of Experiment 1 by establishing independent effects of search difficulty. These search difficulty effects were apparent in later and stronger dilation in the less efficient search condition. Although classification for search difficulty based on features of the pupil response was less accurate, these were robust indicators of search load for almost all participants nonetheless.

### Stimulus-locked versus modeled pupil responses

Critically, in Experiments 1 and 2, there were no interactions between luminance (of the background) and the measures of memory load or search demands on task performance and pupil responses. Thus, at least under the range of static luminance values used in the present study, the load- or difficulty-related pupil response was robust. Echoing these results, we were able to predict both search difficulty and memory load in a particular trial successfully (with better than chance accuracy) from estimates derived from the stimulus-locked data and from continuous modeling of the measured pupil data through impulse functions with the online algorithm. All in all, the variables that best predicted loads or demands were very similar for conventionally calculated time-locked pupil responses and for the algorithmically computed pupil responses. Lending further credence to the validity of the online algorithm, the modeled time points of the attentional pulses were mostly good reflections of the temporal onsets of the stimuli. Admittedly, however, stimulus onsets were sometimes projected a little too far back in time relative to the onsets of the encoding and retrieval displays of the memory task, or they were projected a little after the factual stimulus onset in the VWM task (of Experiment 2). Obviously, the pulses were thus not always mere reflections of the stimulus onsets. This could be due to mere computational reasons—for example, sample window size for the reconstruction of the modeled pupil response. However, this could also be due to the fact that processing onset does not always coincide with stimulus onset. With predictable tasks, as were used here, participants can prepare for an upcoming task, and, likewise, participants are free to begin processing awhile after stimulus onset (e.g., Irons et al., 2017). In other words, our analyses that used the stimulus onsets as ground truth were perhaps too conservative, and factual processing onsets were perhaps not always synchronized with stimulus onsets, but the solution of this issue is beyond the scope of the present research and requires further data.

Moreover, comparing Figures 3C and 4A, Figures 5C and 6A, and Figures 5F and 6B, one can also see some differences in the overall shapes of the time-locked and the algorithmically derived pupillary responses. However, the modeled pupil dilation data also showed clear resemblances to the stimulus-locked data and had similar classification scores. Moreover, the averaged modeled traces often indicated larger differences between conditions than the stimulus-locked data. For example, pupil traces of the two search difficulty conditions in Experiment 2 were significantly different for a larger sequence of consecutive samples than in the stimulus-locked pupil responses.

## Conclusions

In conclusion, the pupil response in general and its modeling by an online algorithm were validated as useful indicators of cognitive load and of search difficulty by several experimental effects of manipulating the corresponding variables as a benchmark. As such, the use of continuous tracking of performance demands by an online algorithm that operates without prior knowledge of task onsets is a promising approach.
